# Pertussis in an adult patient

**DOI:** 10.1590/0037-8682-0439-2024

**Published:** 2025-03-31

**Authors:** Gláucia Zanetti, Elcio Bakowski, Bruno Hochhegger, Edson Marchiori

**Affiliations:** 1 Universidade Federal do Rio de Janeiro, Rio de Janeiro, RJ, Brasil.; 2 Hospital Vivalle - São José dos Campos, SP, Brasil.; 3 University of Florida, Gainesville, FL, USA.

**FIGURE 1:**
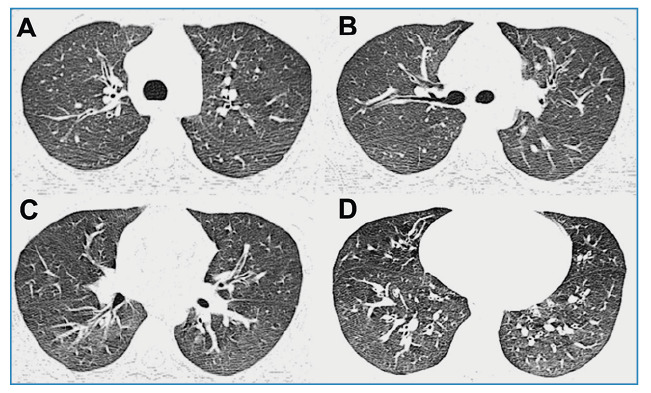
Axial CT images (lung window) showing diffuse thickening of the bronchial walls in a 17-year-old male with pertussis. **CT:** Computed tomography

A 17-year-old boy presented with paroxysmal cough, dyspnea, and vomiting persisting for four weeks. Similar symptoms were observed in the patient’s father and brother. Computed tomography of the chest revealed diffuse thickening of the bronchial wall (Figure 1). Blood tests revealed the presence of *Bordetella pertussis* toxin and immunoglobulin G titer levels of 106.8 IU/mL, leading to the diagnosis of pertussis. The patient was prescribed azithromycin, and an improvement in his symptoms was reported. No coughing or chest pain was reported 8 weeks later.

Pertussis (whooping cough) is a highly contagious respiratory tract infection caused by *B. pertussis.* It is associated with significant morbidity and mortality, particularly in infants. Pertussis is characterized by the presence of an acute cough that often becomes persistent. It is classically associated with coughing, paroxysms, inspiratory whooping, and post-tussive vomiting. The presence of whooping or post-tussive vomiting is suggestive of the diagnosis of pertussis in adult patients with acute or subacute cough; however, the lack of paroxysmal cough or the presence of fever rules out this diagnosis. Post-tussive vomiting is a less useful clinical diagnostic indicator in children^1^ ^-^ ^3^.

Pertussis can be diagnosed through direct detection of the bacterium via culture and polymerase chain reaction or serological methods in adolescents and adults. The primary histopathological findings include tracheitis, bronchitis, and necrotizing bronchiolitis. Complications can arise from the mechanical effects of persistent coughing, including rib fractures, hernias, incontinence, and back pain in adult patients. Other complications include infections, such as sinusitis, otitis media, and pneumonia^1^ ^-^ ^3^.
